# Thyroid Function and the Risk of Prediabetes and Type 2 Diabetes

**DOI:** 10.1210/clinem/dgac006

**Published:** 2022-02-05

**Authors:** Oscar H Roa Dueñas, Anna C Van der Burgh, Till Ittermann, Symen Ligthart, M Arfan Ikram, Robin Peeters, Layal Chaker

**Affiliations:** Department of Epidemiology, Erasmus MC University Medical Center, Rotterdam, the Netherlands; Department of Epidemiology, Erasmus MC University Medical Center, Rotterdam, the Netherlands; Department of Internal Medicine, Erasmus MC University Medical Center, Rotterdam, the Netherlands; Institute for Community Medicine, University Medicine Greifswald, Greifswald,Germany; DZHK (German Center for Cardiovascular Research), Partner Site Greifswald, Greifswald, Germany; Department of Epidemiology, Erasmus MC University Medical Center, Rotterdam, the Netherlands; Department of Epidemiology, Erasmus MC University Medical Center, Rotterdam, the Netherlands; Department of Epidemiology, Erasmus MC University Medical Center, Rotterdam, the Netherlands; Department of Internal Medicine, Erasmus MC University Medical Center, Rotterdam, the Netherlands; Department of Epidemiology, Erasmus MC University Medical Center, Rotterdam, the Netherlands; Department of Internal Medicine, Erasmus MC University Medical Center, Rotterdam, the Netherlands

**Keywords:** thyroid-stimulating hormone, diabetes mellitus, systematic review, meta-analysis, thyroid disease, free thyroxine

## Abstract

**Context:**

Thyroid hormones are important regulators of glucose metabolism, and studies investigating the association between thyroid function and type 2 diabetes incidence have shown conflicting results.

**Objective:**

We aimed to combine the evidence from prospective studies addressing the association between thyroid function and type 2 diabetes risk.

**Methods:**

We systematically searched in Embase, Medline (Ovid), Web of Science, Cochrane, and Google Scholar for prospective studies assessing the association of thyroid function and incident type 2 diabetes. Data extraction was performed using a standardized protocol by 2 independent reviewers. We assessed study quality using the Newcastle-Ottawa Scale and pooled hazard ratios (HRs) and 95% CI using random-effects models.

**Results:**

From the 4574 publications identified, 7 met our inclusion criteria and were included in the qualitative synthesis. Six publications were included in the meta-analysis. Studies assessed hypothyroidism (6 studies), hyperthyroidism (5 studies), thyrotropin (TSH) in the reference range (4 studies), and free thyroxine (FT4) in the reference range (3 studies) in relation to incident type 2 diabetes. The pooled HR for the risk of type 2 diabetes was 1.26 (95% CI, 1.05-1.52) for hypothyroidism, 1.16 (95% CI, 0.90-1.49) for hyperthyroidism, 1.06 (95% CI, 0.96-1.17) for TSH in the reference range, and 0.95 (95% CI, 0.91-0.98) for FT4 in the reference range.

**Conclusion:**

Current evidence suggests an increased type 2 diabetes risk in people with hypothyroidism and lower FT4 levels in the reference range. Further population-based studies are needed to address this association given the limited evidence.

Thyroid hormones are important regulators of metabolism including glucose and protein metabolism (1-3), and an association between thyroid disease and type 2 diabetes has been suggested by some studies (4, 5). Hypothyroidism may result in a reduced metabolic rate, insulin resistance, obesity, and various cardiovascular risk factors, potentially facilitating the incidence of type 2 diabetes (6, 7). On the other hand, thyroid hormone excess has been shown to promote insulin resistance and hyperglycemia, possibly by increasing the glucose absorption in the gastrointestinal tract and glucose hepatic secretion (8).

Several studies have investigated the association between thyroid function and type 2 diabetes with conflicting results (9, 10). A previous systematic review and meta-analysis of three prospective studies showed that thyrotropin (TSH) in the reference range is not associated with type 2 diabetes (11). However, this study assessed only participants with TSH in the reference range and did not explore the association using other measures of thyroid function, such as free thyroxine (FT4) (11). Also, Biondi et al (7) recently performed a comprehensive qualitative systematic review on the link between thyroid dysfunction and type 2 diabetes with a special focus on the underlying mechanisms. The evidence concerning the association of thyroid disease with type 2 diabetes and prediabetes has not previously been aggregated in a quantitative manner. Given the inconsistencies in results from previous studies and the differences across sample characteristics, a systematic review and meta-analysis can provide a comprehensive synthesis of the evidence with an increased sample size and would help in gaining a better understanding of the relation between thyroid function and the incidence of type 2 diabetes, as well as on the potential influence of factors such as the follow-up period and the age of the participants.

In the present systematic review, we aimed to identify and summarize the literature investigating the effect of thyroid function on the incidence of prediabetes and type 2 diabetes. In the subsequent meta-analysis, we aimed to aggregate data on the association between thyroid hormones and type 2 diabetes in prospective cohort studies.

## Methods

### Study Search and Identification

We conducted a systematic literature search to identify prospective cohort studies investigating the association between thyroid function and prediabetes and type 2 diabetes. The systematic review was performed in accordance with the Preferred Reporting Items for Systematic Reviews and Meta-Analyses (PRISMA) guidelines (12). The databases of Embase, Medline (Ovid) (which includes PubMed), Web of Science, Cochrane, and Google Scholar were searched by a trained medical librarian (W.M.B.) from inception through November 27, 2018, without any restriction for language or date (Supplemental Material) (13).

### Eligibility Criteria

We searched for published prospective cohort studies that met the following criteria: (i) baseline thyroid function measurement in participants aged 18 years and older, (ii) prospective assessment of prediabetes or type 2 diabetes as outcomes, and (iii) evaluation of the association between (altered) thyroid function and type 2 diabetes, providing a measure of this association with a risk ratio, odds ratio, or hazard ratio (HR). We excluded studies that used first-generation TSH assays and those that included pregnant women in their sample and/or diabetes gravidarum as an outcome. We also excluded studies that selectively studied patients with thyroid disease or individuals taking thyroid function–altering medication. We included studies in which a proportion of participants were taking thyroid function–altering medication and planned a sensitivity analysis excluding those studies.

### Study Selection

Either of 2 pairs of reviewers (L.C. and S.L. or A.C.B. and O.R.D.) independently screened the titles and abstracts of the search results to determine whether they satisfied the eligibility criteria. Full texts of potentially relevant studies were independently evaluated for inclusion by 2 reviewers. Potential disagreements were discussed and if no consensus was reached, a third reviewer was consulted (R.P.). In addition, the reference lists of all included studies and previously published (systematic) reviews concerning our topic were screened to identify potential eligible studies that were missed in the previous searches.

### Data Extraction and Quality Assessment

A standardized, predefined data extraction form was used to extract information from the full texts of the included studies, including study design, study and participant characteristics, exposure and outcome information, and information about possible confounders and other relevant variables. Two reviewers (A.C.B. and O.R.D.) independently extracted data using the standardized data extraction form. The quality of the included studies was assessed by the 2 independent reviewers as well, using the Newcastle-Ottawa Scale ranging from 0 to 9 (14). In case of any disagreement, a third reviewer was consulted (L.C. or R.P.). Further necessary information on primary outcomes that was not part of the publication was requested from the authors.

### Statistical Analyses

We extracted HRs with the 95% CIs from the studies (9-11, 15-17). We used the DerSimonian and Laird random-effects method (18) to calculate the pooled estimates and 95% CIs of the association between thyroid hormones (TSH in the normal range and FT4 in the normal range) or thyroid disease (hypothyroidism and hyperthyroidism) with type 2 diabetes (or prediabetes).

We evaluated the heterogeneity across studies using the Cochrane *Q* test and the *I*² index (19, 20). We planned 2 additional analyses. First, we categorized studies based on the follow-up to examine whether follow-up time affects our association. Because 25% of people with prediabetes develop type 2 diabetes in a period of 3 to 5 years (21), we decided to stratify the analyses to compare the studies that have a follow-up period of less than 5 years with studies that have a follow-up period of 5 years or longer. Second, we categorized studies by the average age of the participants, comparing those studies of participants who were, on average, 45 years or older with those whose participants were younger than 45 years; and studies with a mean age older and younger than 65 years. Also, we performed the following post hoc analyses: We excluded cohorts with aberrant reference ranges (eg, due to iodine deficiency at the population level) (22). Potential publication bias was evaluated statistically with the Egger test and using funnel plots (23). All statistical analyses were performed using the package “meta” (24) in R statistical software (25) version 4.0.2.

## Results

### Study Selection

**Figure 1.**
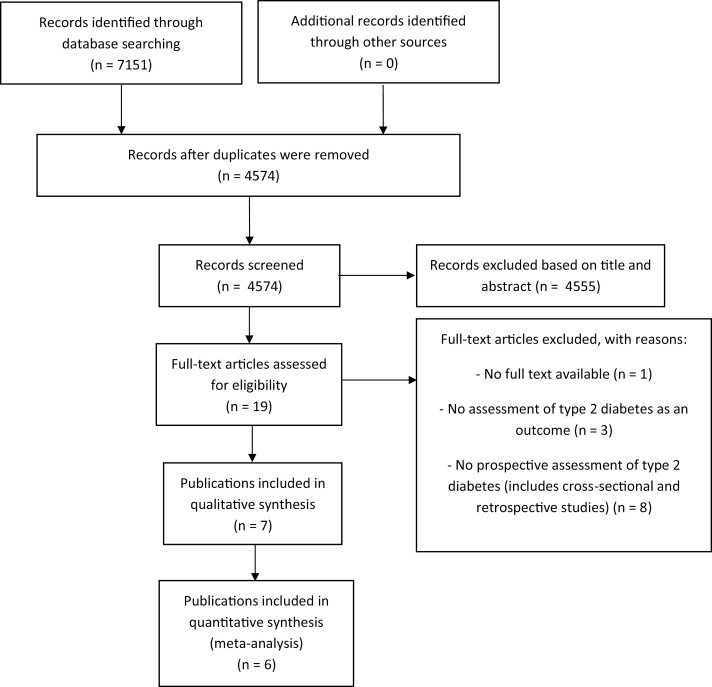
Flowchart.

We identified 7151 reports, and after removing duplicates, 4574 remained. Based on title and abstract, we excluded 4555 reports because they were not related to the association between (subclinical) thyroid (dys)function and prediabetes or type 2 diabetes. Of the remaining 19 publications, 7 met our inclusion criteria after full-text screening. From these, one publication did not provide necessary estimates even after requesting the data (26), and thus was included only in the qualitative synthesis (Fig. 1). Two authors provided additional information. Chaker et al (15) provided the estimates in hypothyroidism, and in TSH in the reference range without natural logarithm transformation. Ittermann et al (9) provided the estimates of TSH and FT4 in the reference range and overt thyroid disease in the studies of SHIP and INTER99, and using slightly different measurements (larger follow-up) in the SHIP cohort compared to the ones used in the original publication. We could not quantitatively summarize the association of thyroid function with prediabetes because this was assessed by only one study (10).

### Study Characteristics

We included 7 publications that used data from 8 study samples, with a total of 176 788 participants with 13 261 cases of incident type 2 diabetes (Table 1). Three of the 8 studies were population-based prospective cohort studies; 1 was a cohort study conducted in patients with atherosclerotic vessel disease or marked risk factors for atherosclerosis (11); 1 was a population-based, randomized controlled trial that investigated the effects of lifestyle interventions on cardiovascular disease (CVD; INTER99 cohort) (9, 27); 2 examined a sample of individuals with hypothyroidism or hyperthyroidism, respectively, matched with controls from the general population (based on Danish health registers) (16, 17); and 1 evaluated a cohort of statin users, matched with a cohort of nonstatin users from the general population (26). All studies were from Europe and Asia. The mean age of participants across studies ranged from 41.2 to 67.0 years and the mean follow-up duration varied from 3.4 to 11.2 years. In addition, 1 study contained information on the progression from prediabetes to type 2 diabetes with 358 type 2 diabetes cases among a sample of 1137 participants with prediabetes (analyses for TSH and FT4 in the reference range) (15) and 1 examined prediabetes as an outcome (10). Six studies informed about hypothyroidism with a total of 158 091 participants and 11 894 cases of incident type 2 diabetes. Furthermore, 1 study provided information on subclinical hypothyroidism analyzing 59 597 participants with 8076 cases of incident type 2 diabetes (26). Hyperthyroidism was examined by 5 studies, with a total of 148 684 participants and 11 154 incident cases of type 2 diabetes. No study provided information on subclinical hyperthyroidism. Four studies examined TSH in the normal range (9, 11, 15) and 3 of these also assessed FT4 in the reference range (9, 15).

**Table 1.** Characteristics of included studies

Source	Country	Study baselineyear	Design	Study sample	Total No.	Age, mean	Incident cases of type 2 diabetes	Hypothyroidism	Subclinical hypothyroidism	Euthyroid participants	Hyperthyroidism
							No. (%)	No. (%)	No. (%)	No. (%)	No. (%)
Brandt, 2013	Denmark	1996-2008	Prospective nationwide registry-based cohort study	Sample of hyperthyroid individuals, and matched sample of controls (1:4) from general population	13 155	67	1078(8.19)	NR	NR	10 524(80.0)	2631(20.0)
Thvilum, 2013	Denmark	1996-2008	Prospective nationwide registry-based cohort study	Sample of hypothyroid individuals, and matched sample of controls (1:4) from general population	14 110	58	1020(7.23)	2822(20.0)	NR	11 288(80.0)	NR
Gronich, 2015	Israel	2004-2005	Prospective registry-based cohort study	Cohort of statin users, and matched cohort from general population	59 597	63.8^*c*^	8076(13.55)	4780(8.02)^*d*^	192(0.32)	54 141 (90.85)	484(0.81)
Chaker, 2016	Netherlands	1997-2008	Prospective cohort study	General population	8452	64.6	798(9.44)	62(0.73)	NR	7310 (86.49)	NR
Chang, 2017	Taiwan	1996-2004	Prospective registry-based cohort study	General population	68 846^*a*^	41.2	1551(2.25)	1101(1.60)	NR	65 499 (95.14)	2246(3.26)
Ittermann, 2018 (SHIP)^*b*^	Germany	1997-2001	Prospective cohort study	General population	3057	48.1	287(9.39)	73(2.39)	NR	2743 (89.73)	241(7.88)
Ittermann, 2018 (INTER99)^*b*^	Denmark	1999-2001	Randomized controlled trial of lifestyle interventions on IHD	General population	4029	46.4	162(4.02)	80(1.99)	NR	3907 (96.97)	42(1.04)
de Vries, 2019	Netherlands	2003-2015	Prospective cohort study	Patients with atherosclerotic vessel disease or marked risk factors for atherosclerosis	5542	56.1	289(5.21)	NR	NR	5542(100)	NR
Source	Mean follow-up duration, year	Reference range		Hypothyroidism	Hyperthyroidism	TSH in reference range	FT4 in reference range	FT3	Relevant adjustments		
		TSH	FT4								
Brandt, 2013	6	NR	NR	NR	HR, Singletons 1.46 (1.16-1.84)	NR	NR	NR	Charlson score. Matching performed by age and sex between participants with thyroid disease and controls		
Thvilum, 2013	6	NR	NR	HR, Singletons 1.40 (1.11-1.77)	NR	NR	NR	NR	Charlson score. Matching performed by age and sex between participants with thyroid disease and controls		
Gronich, 2015	5-7	NR	NR	RR, Statin users 2.06 (1.42-2.99); RR, Statin nonusers 1.66 (1.05-2.64)^*h*^	RR, Statin users 1.27 (0.75-2.13); RR, Statin nonusers 0.52 (0.19-1.40)	NR	NR	NR	Age, sex, ethnicity, obesity, smoking, serum glucose, LDL, HDL, and triglyceride levels at baseline, history of hypertension, and medication use		
Chaker, 2016	7.9	0.4-4.0 mIU/L	11-25 pmol/L	HR, 1.48 (0.73-2.98)	NR	HR, 1.11 (1.02-1.22)^*g*^	HR, 0.94 (0.90-0.98)	NR	Sex, age, smoking, cohort, fasting serum glucose levels, fasting serum insulin measurements, systolic blood pressure, diastolic blood pressure, blood pressure–lowering medication, HDL cholesterol, and BMI		
Chang, 2017	3.4^*e*^	0.47-5 μU/mL	NA	HR, 0.96 (0.64-1.44)	HR, 1.05 (0.80-1.39)	NA	NA	NA	Sex, age group, education level, smoking, drinking, and obesity		
Ittermann, 2018 (SHIP)^*b*^	11.2	0.25-2.12 mIU/L	8.3-18.9 pmol/L	HR, 0.98 (0.53-1.81)	HR, 0.96 (0.65-1.42)	HR, 1.12 (0.78-1.60)	HR, 0.93 (0.88-0.99)	IRR, 1.21 (1.16-1.27)^*f*^	Age, sex, BMI, and smoking		
Ittermann, 2018 (INTER99)^*b*^	5.3	0.30-4.00 mIU/L	12-18.9 pmol/L	HR, 1.48 (0.54-4.02)	HR, 0.56 (0.08-4.02)	HR, 0.84 (0.66-1.08)	HR, 1.02 (0.92-1.13)	NR	Age, sex, and BMI		
de Vries, 2019	5.6	0.35-5.0 mIU/L	NR	NA	NA	HR, 1.07 (0.95-1.22)	NR	NR	Age, sex, current smoking, total and HDL cholesterol, and triglycerides		

Abbreviations: BMI, body mass index; FT3, free triiodothyronine; FT4, free thyroxine; HDL, high-density lipoprotein; HR, hazard ratio; IHD, ischemic heart disease; IRR, incidence rate ratio; LDL, low-density lipoprotein; NA, not applicable; NR, not reported; RR, risk ratio; TSH, thyrotropin.

^*a*^Number of participants in longitudinal analyses.

^*b*^The SHIP and INTER99 cohorts were included in the publication by Ittermann et al (9).

^*c*^Weighted mean age at baseline.

^*d*^Does not include subclinical hypothyroidism, and includes treated and untreated hypothyroid participants.

^*e*^Weighted mean follow-up across TSH level groups.

^*f*^Adjusted for age and sex.

^*g*^Adjusted for age, sex, cohort, smoking, and BMI.

^*h*^RR for hypothyroidism not treated with thyroid hormone replacement.

Three studies investigated the association between overt thyroid disease and type 2 diabetes using matched samples: Two of the studies matched hypothyroid or hyperthyroid individuals with controls from the general population (16, 17), and the other study examined the association of thyroid disease and type 2 diabetes in a matched sample of statin and nonstatin users (26). Among the studies that examined hypothyroidism and that did not use a matching design, the percentage of hypothyroidism ranged between 0.73% and 2.39% (9, 10, 15). The percentage of hyperthyroidism in studies that did not use a matching design ranged between 1.04% and 7.88% (9, 10). The studies showed similar scores on the Newcastle-Ottawa Scale assessment scale ranging from 6 to 9.

### Qualitative Synthesis

Among the 6 studies examining hypothyroidism and type 2 diabetes, 2 showed an increased risk of type 2 diabetes after the diagnosis of hypothyroidism (17, 26), whereas the others did not find a statistically significant association (9, 10, 15). Interestingly, Chang et al (10) reported that hypothyroid participants had an increased risk for prediabetes, but not for type 2 diabetes. Hyperthyroidism, analyzed by 5 studies, was not related to type 2 diabetes (inconsistent direction of effects and nonstatistically significant associations), except for the study by Brandt et al (16), in which hyperthyroidism had a positive association with the risk of type 2 diabetes. Importantly, among the studies examining thyroid disease, 3 included dispensed prescriptions of (anti-)thyroid medication and/or International Classification of Diseases Revision 8 (ICD-8), ICD-9, and ICD-10 codes as sources to define thyroid disease (16, 17, 26). TSH in the reference range was investigated by 4 studies (9, 11, 15). Only 1 found a positive significant association between TSH in the reference range and type 2 diabetes (15), whereas the others found no statistically significant association (9, 11). Three studies assessed FT4 in the reference range, with 2 reporting that participants with higher levels of FT4 had a lower risk of type 2 diabetes (9, 15), and 1 describing a nonstatistically significant association (9). Furthermore, the study by Ittermann et al (9) (SHIP) was the only one to examine free 3,5,3′-triiodothyronine (FT3). In this study, FT3 levels were positively associated with type 2 diabetes (9) (Table 1).

### Meta-Analysis: Thyroid Function and Type 2 Diabetes

**Figure 2.**
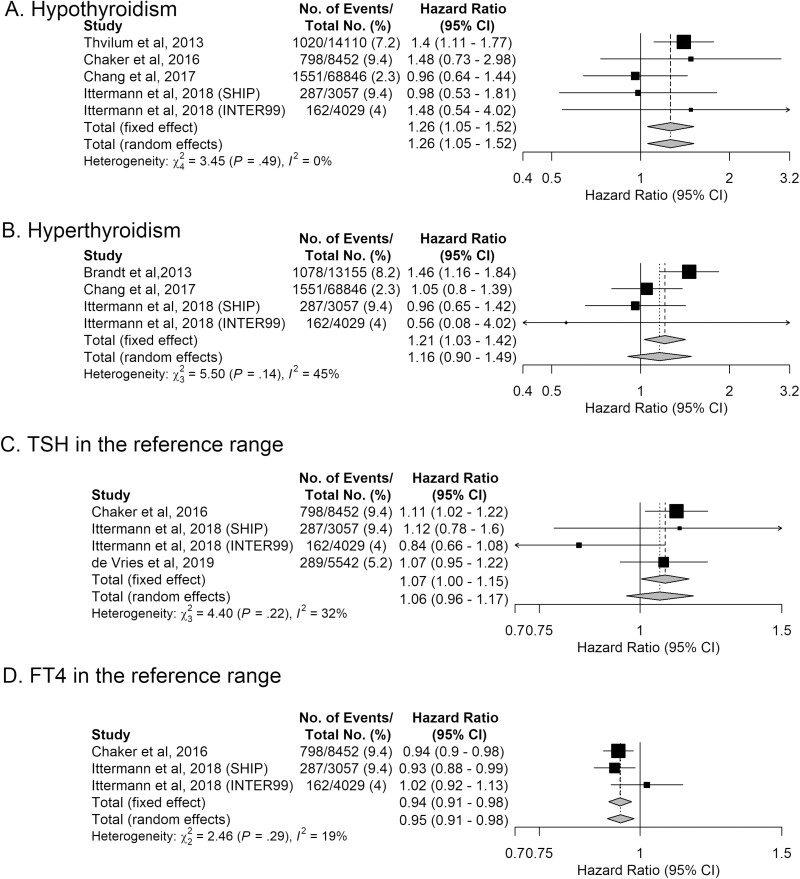
Forest plots for the association between thyroid disease or thyroid function and incident type 2 diabetes. A, Hypothyroidism and type 2 diabetes. B, Hyperthyroidism and type 2 diabetes. C, Thyrotropin (TSH) in the reference range and type 2 diabetes. D, Free thyroxine (FT4) in the reference range and type 2 diabetes. The diamonds represent the pooled hazard ratios.

We examined the association between thyroid disease or thyroid function and type 2 diabetes using random-effects models. The pooled HR for the association between hypothyroidism and the risk of incident type 2 diabetes was 1.26 (95% CI, 1.05-1.52) without heterogeneity (*I*^2^ of 0%; *P* = .49) (Table 2, Fig. 2A). For hyperthyroidism, the results showed an association with the risk of type 2 diabetes, although not statistically significant, with a pooled HR of 1.16 (95% CI, 0.90-1.49) and moderate heterogeneity (*I*^2^ of 45.50%; *P* = .14) across the 4 studies (see Table 2, Fig. 2B). We further pooled the HR across 4 studies with TSH in the reference range and found some, but not statistically significant evidence for a positive association with the risk of developing type 2 diabetes (HR: 1.06; 95% CI, 0.96-1.17) (*I*^2^ of 31.90%; *P* = .22) (Table 3, Fig. 2C). There was a decreased risk of type 2 diabetes with higher levels of FT4 within the reference range (HR: 0.95; 95% CI, 0.91-0.98). The latter analyses were performed by pooling only 3 studies, with an *I*^2^ of 18.80% and a *P* value for heterogeneity of .29, representing low heterogeneity (see Table 3, Fig. 2D).

**Table 2.** Association between thyroid disease and type 2 diabetes

	Hypothyroidism				Hyperthyroidism			
	Pooled HR	No. of studies	*P* for heterogeneity	*I* ^2^, %	Pooled HR	No. of studies	*P* for heterogeneity	*I* ^2^, %
Main results								
Random effects	1.26 (1.05-1.52)	5	.49	0	1.16 (0.90-1.49)	4	.14	45.5
Fixed effects	1.26 (1.05-1.52)	5	.49	0	1.21 (1.03-1.42)	4	.14	45.5
Additional analyses								
Excluding SHIP								
Random effects	1.30 (1.07-1.57)	4	.44	0	1.23 (0.91-1.66)	3	.14	48.7
Fixed effects	1.30 (1.07-1.57)	4	.44	0	1.27 (1.06-1.51)	3	.14	48.7
Follow-up ≥ 5 year								
Random effects	1.36 (1.11-1.66)	4	.74	0	1.19 (0.82-1.74)	3	.14	49.9
Fixed effects	1.36 (1.11-1.66)	4	.74	0	1.30 (1.07-1.58)	3	.14	49.9
Including those < 65 year								
Random effects	1.23 (1.00-1.52)	4	.36	7.6	1.01 (0.81-1.27)	3	.78	0
Fixed effects	1.25 (1.04-1.51)	4	.36	7.6	1.01 (0.81-1.27)	3	.78	0

Additional information about sensitivity analyses in hypothyroidism and hyperthyroidism: 1. There were not enough studies to perform the analysis for the mean age of 65 years or older and for the mean age of younger than 45 years. 2. The sensitivity analysis of studies with mean age of 45 years or older is the same as the analysis of studies with a follow-up of 5 or more years.

Abbreviation: HR: hazard ratio.

**Table 3.** Association between thyroid hormones and type 2 diabetes

	TSH in reference range				FT4 in reference range			
	Pooled HR	No. of studies	*P* for heterogeneity	I², %	Pooled HR	No. of studies	*P* for heterogeneity	*I*², %
Main results								
Random effects	1.06 (0.96-1.17)	4	.22	31.9	0.95 (0.91-0.98)	3	.29	18.8
Fixed effects	1.07 (1.00-1.15)	4	.22	31.9	0.94 (0.91-0.98)	3	.29	18.8
Additional analyses								
Excluding SHIP								
Random effects	1.05 (0.93-1.18)	3	.11	54.0	0.97 (0.90-1.04)	2	.15	51.7
Fixed effects	1.07 (1.00-1.15)	3	.11	54.0	0.95 (0.91-0.99)	2	.15	51.7
Age, year								
Including those < 65 year								
Random effects	1.01 (0.86-1.18)	3	.20	37.4	0.96 (0.88-1.05)	2	.13	57.2
Fixed effects	1.03 (0.92-1.14)	3	.20	37.4	0.95 (0.90-1.00)	2	.13	57.2

Additional information about sensitivity analyses in TSH and FT4 in the reference range: 1. There was only one study with mean age of 65 years or older. 2. The sensitivity analyses for studies stratified by mean age younger than 45 vs 45 years or older and analyses based on the follow-up were not possible because all studies have a mean age of 45 years or older and a follow-up of 5 or more years.

Abbreviations: FT4, free thyroxine; HR, hazard ratio; TSH, thyrotropin.

We performed additional analyses, first by excluding the SHIP cohort from the analyses of thyroid disease and thyroid function reference ranges because of aberrant reference ranges (9). Overall, we obtained similar results compared to the main analyses, except for the association between FT4 in the reference range and the risk of type 2 diabetes, which became nonstatistically significant (see Tables 2 and 3). Second, after stratifying based on follow-up, the effect estimate of the association became stronger when we included only studies with a follow-up period longer or equal to 5 years in the analyses of hypothyroidism but not for hyperthyroidism (see Table 2). In addition, we planned to study the association of thyroid disease and type 2 diabetes in studies with a follow-up period of less than 5 years, but we could not investigate this because only 1 study had a follow-up in this time frame (10). It was also not possible to perform the analyses stratified by follow-up using the TSH and FT4 in the reference range because all studies had a follow-up period of more than 5 years. Finally, we planned to stratify the analyses based on the mean age of participants (age older and younger than 45 years, and older and younger than 65 years). However, owing to limited data, it was only possible to examine studies with participants who were, on average, older than 45 years (same studies as in the analyses of follow-up > 5 years), and participants younger than 65 years. We found no evidence for an association in the latter analyses (see Tables 2 and 3). Sensitivity analyses excluding studies of patients receiving thyroid function–altering medication were not performed because of limited data.

### Evaluation of Publication Bias

Although based on a limited number of studies, visual inspection of the funnel plots for the associations between thyroid disease (hypothyroidism and hyperthyroidism) or thyroid function in the reference range (TSH and FT4) with the risk of type 2 diabetes did not show evidence for publication bias (Supplementary Fig. 1) (13). Similarly, the Egger test showed no signs of publication bias for any of the associations (hypothyroidism and type 2 diabetes: *P* = .66, hyperthyroidism: *P* = .39, TSH in the reference range: *P* = .42, FT4 in the reference range: *P* = .38).

## Discussion

In this systematic review and meta-analysis, we report that hypothyroidism is associated with an increased risk of developing type 2 diabetes, and higher FT4 in the reference range with a decreased risk of developing type 2 diabetes. Hyperthyroidism and TSH in the reference range were not statistically significantly associated with type 2 diabetes.

Based on the effects of thyroid hormones on the metabolism of carbohydrates, lipids, and insulin secretion (3), the association between thyroid function and type 2 diabetes has long been hypothesized. In addition, thyroid hormones may cause pathological conditions that in turn act as risk factors for type 2 diabetes, such as an increased body mass index and impaired insulin secretion (6, 28). For example, an association between low FT4 and insulin resistance has been reported in euthyroid participants (29), and low FT4 levels have been found to be related to metabolic syndrome (30). In this study, after pooling the findings of 3 studies, we describe a relation between lower levels of FT4 within the reference range and a higher risk of developing type 2 diabetes (9, 15). FT3 was assessed in only one study (SHIP), and the results suggested that baseline FT3 levels are positively associated with the incidence of type 2 diabetes (9). However, we were not able to evaluate this association in our meta-analysis because of limited data.

Regarding overt thyroid disease, hypothyroidism has been related to insulin resistance and alterations in glucose metabolism (28), which might suggest that hypothyroidism could increase the incidence of type 2 diabetes. In fact, a recent review described that hypothyroid disease may cause insulin resistance possibly through an alteration of GLUT4 translocation and the effects of leptin, and an elevation of free fatty acids (7). Interestingly, hyperthyroidism may also cause a hyperglycemic state, through an increase in the GLUT2 transporters in the liver, lipolysis, and nonoxidative glucose disposal, which in turn may lead to an increase in hepatic glucose output and therefore, a hyperglycemic state (28). Hyperthyroidism is additionally associated with an increase in glucose use in the skeletal muscle and reduced glycogen synthesis (28, 31). The 2 latter mechanisms may result in a hypoglycemic state and thus counteract the pathways that lead to hyperglycemia. This hypothesis can be a possible explanation for the absence of an association between hyperthyroidism and type 2 diabetes in our analyses. Another explanation to be considered is that participants with thyrotoxicosis may receive treatment at an early phase, limiting the potential to observe an association between hyperthyroidism and type 2 diabetes in prospective studies. Furthermore, time is a relevant variable to take into account when studying thyroid disease and type 2 diabetes. The effect estimate of hypothyroidism became stronger after we excluded studies with a follow-up period of less than 5 years, suggesting that the effect may need time to emerge. In fact, previous studies have described that it may take around 5 years to develop type 2 diabetes from prediabetes (21). Importantly, the only study excluded because of a short follow-up also had the participants with the youngest mean age (41.2 y at the study population baseline) (10). Considering that the prevalence of type 2 diabetes is lower in individuals younger than 50 years (32), this might also explain why the effect became stronger after excluding the study by Chang et al (10). However, future studies should examine the association between thyroid function/thyroid disease and type 2 diabetes in young adults, as thyroid disease was shown to be related to higher risk of type 2 diabetes in participants aged 18 to 39 years in a recent cohort study (33). Also, another important factor to consider is sex because thyroid dysfunction is more common in women (7) and type 2 diabetes is more common in men (34). All studies in this meta-analysis were adjusted for sex (Thvilum et al [17] and Brandt et al [16] matched exposure groups by sex).

Two previous mendelian randomization studies have been performed with conflicting results (35, 36). The first by Bos et al (35) found no association between TSH/FT4 with type 2 diabetes, suggesting that the association reported by traditional observational studies could be due to reverse causation or unmeasured confounding (35). However, Bos and colleagues did find an association between genetic variants related to thyroid metabolism and insulin resistance (35), supporting a potential role of thyroid function in the pathogenesis of type 2 diabetes. A more recent mendelian randomization study by Kuś et al (36) found no association between thyroid hormones and type 2 diabetes in their main analyses, but when pleiotropic instruments were excluded, they observed that greater levels of TSH were statistically significantly associated with lower risk of type 2 diabetes. More research is needed to reconcile the differences between observational studies and mendelian randomization studies by exploring potential underlying mechanisms (eg, mediation analyses) and through an increase in genetic instruments for hypothyroidism and hyperthyroidism as well as TSH and FT4 (eg, larger explained genetic variability).

A strength of the present meta-analysis includes the assessment of the role both of FT4 in the reference range and overt thyroid disease in relation to the risk of incident type 2 diabetes. We performed a literature search in numerous electronic databases with minimal restrictions to find as many as possible of the available studies about this topic in the literature. However, there are some limitations. First, the number of studies that met the inclusion criteria was low, showing a lack of literature on this topic, which did not allow for more robust and in-depth investigation of the association. Second, we cannot completely rule out an effect of heterogeneity and publication bias given the small number of studies on this topic. Third, there were differences in the mean age of participants across studies, ranging from 41.2 to 67.0 years. Furthermore, only 2 studies included older participants (age ≥ 65 years) (15, 16), limiting the possibility of drawing firm conclusions for this age group. Fourth, it is possible that hypothyroid and hyperthyroid participants received treatment after the baseline measure. However, it was not possible to assess the effect of thyroid treatment at follow-up on the association between thyroid disease and the risk for type 2 diabetes because of a lack of information in most of the studies included in this meta-analysis. Fifth, the definition of prediabetes in the studies found in our systematic review was either based on fasting glucose levels, no diabetes history, and no antidiabetic medication use (10), or based on World Health Organization guidelines (15). Considering that prediabetes is often defined based on fasting glucose levels or on glucose tolerance (37), it is important to note that impaired glucose tolerance is related to CVD (38), whereas the evidence for impaired fasting glucose and the relation to CVD is mixed (38, 39). Also, FT3 levels are differently related to impaired fasting glucose and to impaired glucose tolerance (40). Unfortunately, we did not have information on T3, thus we were not able to study the different mechanisms by which T3 could affect the risk for prediabetes. Finally, a systematic review and meta-analysis by Han et al (41) of cross-sectional and case-control studies showed a potential link between subclinical hypothyroidism and type 2 diabetes. Owing to a lack of sufficient data, we could not address this association in our study.

### Conclusion

Overall, the present meta-analysis study provides evidence for a long-term association of hypothyroidism and FT4 in the reference range with the incidence of type 2 diabetes, but not for hyperthyroidism or TSH in the reference range. To date, only a few studies have prospectively studied the association between thyroid (dys-)function and the risk of type 2 diabetes, revealing a gap in the literature concerning a topic involving the 2 most common endocrinological disorders. Also, more prospective studies are needed to identify the role of thyroid hormones in individuals with prediabetes because these have the highest risk of developing type 2 diabetes and could be an interesting group to target as part of primary prevention. These studies may inform potential future randomized, controlled trials investigating thyroid function as a modifiable risk factor for the incidence of type 2 diabetes.

## Data Availability

Data sharing is not applicable to this article because no data sets were generated or analyzed during the present study.

## Abbreviations

CVD: cardiovascular disease
FT3: free triiodothyronine
FT4: free thyroxine
HR: hazard ratio
ICD: International Classification of Diseases
TSH: thyrotropin
